# Missed fractures of the greater tuberosity

**DOI:** 10.1186/s12891-018-2225-1

**Published:** 2018-08-31

**Authors:** Umile Giuseppe Longo, Steven Corbett, Philip Michael Ahrens

**Affiliations:** 1 Department of Orthopaedic and Trauma Surgery, Campus Bio-Medico University, Via Alvaro del Portillo, 200, 00128 Rome, Italy; 2 Shoulder Unit, Hospital of St John and St Elizabeth, 60 Grove End Road, London, UK

**Keywords:** Shoulder, Fractures, Greater tuberosity, Management, Occult

## Abstract

**Background:**

Fractures of the greater tuberosity may result from a variety of mechanisms. Missed injury remains a persistent problem, both from a clinical and medico-legal point-of-view. Few studies on this topic are available in the literature. We present the clinical and radiological findings of a consecutive series of 17 patients who were diagnosed and managed with undisplaced greater tuberosity fractures.

**Methods:**

A retrospective study of a consecutive series of 17 patients who sustained an occult greater tuberosity fracture were performed. Patients sustained a traumatic occult greater tuberosity fracture, underwent shoulder radiographs after trauma in 5 days and they were diagnosed as negative by a consultant radiologist. All patients received a standard assessment using MRI (Magnetic Resonance Imaging) scans Each patient was evaluated for arm dominance, trauma history, duration and type of symptoms and post-treatment Oxford Shoulder Score.

**Results:**

At the final follow up the mean OSS (Oxford Shoulder Score) was 38.3 (range 17–46; SD 9.11). Three patients required a glenohumeral joint injection for post-traumatic pain and stiffness and three patients required subacromial decompression for post-traumatic impingement.

**Conclusions:**

Though undisplaced greater tuberosity fracture can be managed non-operatively with good results, patients with persistent post-traumatic shoulder pain, tenderness and limitation of shoulder function warrant investigation with MRI to identify occult fractures. Prompt identification of these fractures can facilitate patient treatment and counselling, avoiding a source of patient dissatisfaction and litigation.

## Background

Fractures of the greater tuberosity may result from a variety of mechanisms. The most common are avulsion injuries such associated with anterior shoulder dislocation, or direct trauma, as might occur in a fall on the shoulder or with hyperabduction and impaction of the greater tuberosity against the surrounding bone structures [1, 2]. These fractures can be misdiagnosed, as radiographs of the shoulder are often insufficient to confirm the diagnosis [2], especially in the case of undisplaced fractures and if the radiographic series does not include an anteroposterior (AP) view with the arm in external rotation [2, 3]. Patients may complain of persistent rotator cuff symptoms and may be referred for further examination. Missed injury remains a persistent problem, both from a clinical and medico-legal point-of-view. Few studies on this topic are available in the literature.

We present the clinical and radiological findings of a consecutive series of 17 patients who were diagnosed and managed with undisplaced greater tuberosity fractures.

## Methods

Few months ago we decided to publish a retrospective study of a consecutive series of patients who sustained an occult greater tuberosity fracture and were managed at our institution between 2006 and 2008. All patients gave written consent to participate in the study. The study was submitted and approved by the ethics committee of “Campus Bio Medico” of Rome.

### Eligibility criteria

Patients were included in the study if (1) they sustained an occult greater tuberosity fracture, (2) they had a traumatic shoulder injury, (3) they underwent shoulder radiographs after trauma in 5 days and they were diagnosed as negative by a consultant radiologist, (4) the treating physician initially managed all the patients as a soft tissue injury, (5) all the diagnoses were made on the basis of an MRI performed within 6 weeks of the initial trauma, and (6) no previous history of shoulder symptoms.

An occult greater tuberosity was defined as the MRI findings of oedema in the greater tuberosity at T2-weighted images associated with a fracture line and/or cortical breach [4]. A crescent or oblique line of decreased signal intensity can be found at T1- or T2-weighted images of patients with greater tuberosity fracture [4].

The indication for MRI was shoulder pain associated with the clinical finding of tenderness on palpation of the greater tuberosity in the presence of negative radiographs.

Patients were excluded from the study if they had (1) associated rotator cuff tear, (2) previous surgery on the affected shoulder, (3) a displaced fracture of the greater tuberosity, (4) Hill-Sachs lesions or evidence of shoulder dislocation, (5) a glenoid rim fracture, (6) no history of trauma.

### Patient demographics

Seventeen patients met the inclusion criteria. 6 patients were managed primarily by the authors, and 11 patients were secondary referred to them. All the patients were initially managed non-operatively. Of the 17 included patients, 16 agreed to participate in the study. 1 patient made a formal complaint against the initial treating physician and he declined to participate in the study. 3 patients agreed to participate in the study, but they moved abroad and were not contactable for final follow-up. Finally 13 patients were analyzed. The dominant arm was involved in 12 patients. Mechanism of index injury and associated shoulder MRI findings are reported in Table 1 for each patient.

**Table 1:** MRI findings for each patient

Patients	Affected/Dominant limb	Duration of follow-up (months)	Interval from initial injury to diagnosis	Referral	Associated injury at MRI	Type of primary injury	Treatment	Complications	Worker claim compensation	OSS
1	R/R	9 months	12 days	Secondary	None	Fall from scaffold	nonoperative, plus 1 glenohumeral joint injection	Stiffness at 6 weeks post-treatment, resolved at the final follow up	No	41
2	L/R	8 months	3 days	Primary	None	Rugby	nonoperative	No	No	46
3	R/R	8 months	67 days	Secondary	Partial thickness supraspinatus tear	Patagonian twister	nonoperative plus 1 glenohumeral joint injection 3 months after injury	Stiffness at 3 months post-treatment, resolved at the final follow up	No	31
4	L/R	8 months	60 Days	Secondary	None	Rugby army	nonoperative plus 1 glenohumeral joint injection 3 months after injury	Stiffness 3 months post-treatment, resolved at the final follow up	No	45
5	R/R	12 months	42 days	Secondary	None	Fall	nonoperative	No	No	31
6	R/R	15 months	28 days	Secondary	None	Direct fall on the right shoulder while cycling	nonoperative	No	No	46
7	R/R	11 months	8 days	Primary	Subscapularis tear	Fall down steps	nonoperative	No	No	Abroad
8	L/R	8 months	8 days	Primary	None	Fall	nonoperative	No	No	Declined
9	R/R	10 months	3 weeks	Primary	None	Fall on ice	nonoperative	No	No	Abroad
10	R/R	44 months	3 months	Secondary	None	Fall playing soccer	nonoperative	Post-traumatic impingement managed with subacromial decompression	No	Abroad
11	R/R	10 months	8 weeks	Secondary	None	Motocycle road traffic accident	nonoperative	No	No	30
12	R/R	46 months	2 months	Secondary	None	Fall while skiing	nonoperative	Post-traumatic impingement managed with subacromial decompression	No	48
13	L/L	6 months	3 months	Secondary	None	Fall while skiing	nonoperative	No	No	17
14	R/R	17 months	3 weeks	Primary	None	Bicycle road traffic accident	nonoperative	No	No	38
15	L/R	18 months	5 weeks	Secondary	None	Bicycle road traffic accident	nonoperative	No	No	44
16	L/R	3 months	1 day	Primary	None	Fall down stairs	nonoperative	No	No	46
17	R/R	20 months	4 months	Secondary	None	Fall from ladder	nonoperative	Post-traumatic impingement managed with subacromial decompression	No	35

### Evaluation

Clinical evaluations were performed at a mean of 15 months (range, 3–44 months) from the diagnosis. Arm dominance, clinical history and post-treatment Oxford Shoulder Score were evaluated for each patient.

### Imaging

Standard radiographs were performed for all patients in anteroposterior projections and a scapular lateral view or an axillary view.

MRI scans consisted of oblique coronal, oblique sagittal, and axial T2-weighted spin-echo MRIs (repetition time, 3200 milliseconds; echo time, 85 milliseconds).

### Functional assessment

The Oxford shoulder score (OSS) was used to evaluate shoulder function. OSS is a patient-based questionnaire used to assess shoulder pain and function. The final score had a range from 12 (least difficulties) to 60 (most difficulties) [5, 6].

### Non-operative management

A sling without pillow in slight internal rotation was used for 4 weeks. The sling was removed during bathing and exercises. Patients performed active elbow flexion and extension and pendular exercises as tolerated from the day of diagnosis [7].

At 4 weeks, the sling was discontinued and active assisted shoulder flexion, extension, abduction, external rotation and internal rotation were commenced. Isometric strengthening was not begun until 6 weeks post-injury, at which point we began rehabilitation of the rotator cuff, deltoid, and scapular stabilizers according to a validated protocol (http://www.moonshoulder.com/impactstudy.html).

## Results

Of the 17 included patients, 16 agreed to participate in the study. 1 patient made a formal complaint against the initial treating physician and he declined to participate in the study. 3 patients were not contactable for final follow-up.

8 of the 17 patients had a sports related injury.

Our average follow up was 16.5 months, with all patients having 6 months or more follow up, except one patient who was fully recovered at 3 months with an OSS of 46. The internal consistency of OSS score was measured by the Cronbach’s alpha, with 0.89 at the preoperative assessment and 0.92 at 6-month follow-up. A coefficient of test-retest reliability of 6.8 was obtained using the Bland and Altman method. A significant correlation was obtained with Constant score, SF36 and Health Assessment Questionnaire Disability Index. The sensitivity to change of the study questionnaire was examined by comparing scores before and 6 months after operation, and it showed that the OSS is sensitive to clinical changes [5, 6]. The OSS in our patients showed good long term clinical results.

An issue which we highlight is that 3 of our patients were not able to attend for the final follow up, and we had to rely on the latest available clinical outpatient follow up result.

After trauma, all patients had pain in elevation and external rotation of the humerus. At diagnosis, all patients had minimal or no displacement of the fracture fragment. All fractures were undisplaced; therefore non-operative management was performed. Of the 16 included patients, OSS data were available for 13 of them. At the final follow up the mean OSS was 38.3 (range 17–46; SD 9.11).

**Fig. 1:**
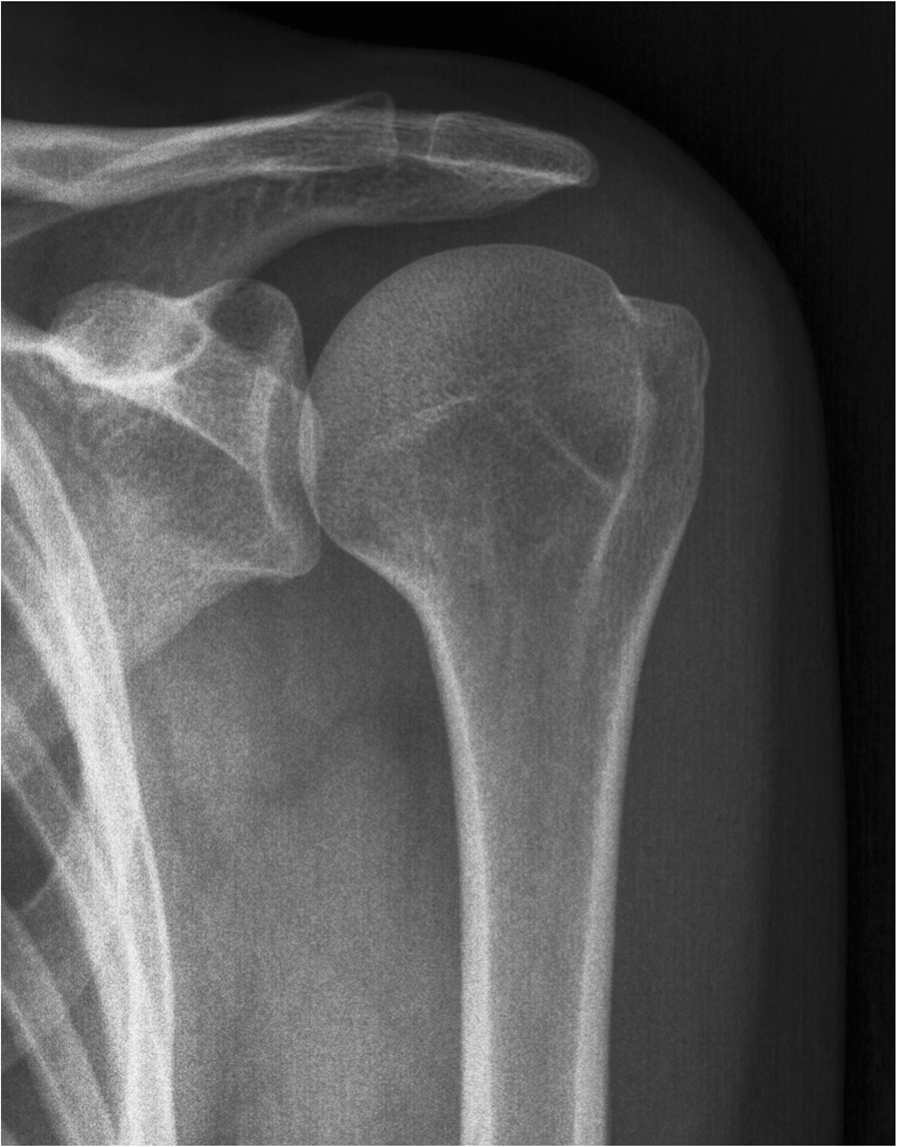
Shoulder radiographs of a patient with undisplaced greater tuberosity fracture

**Fig. 2:**
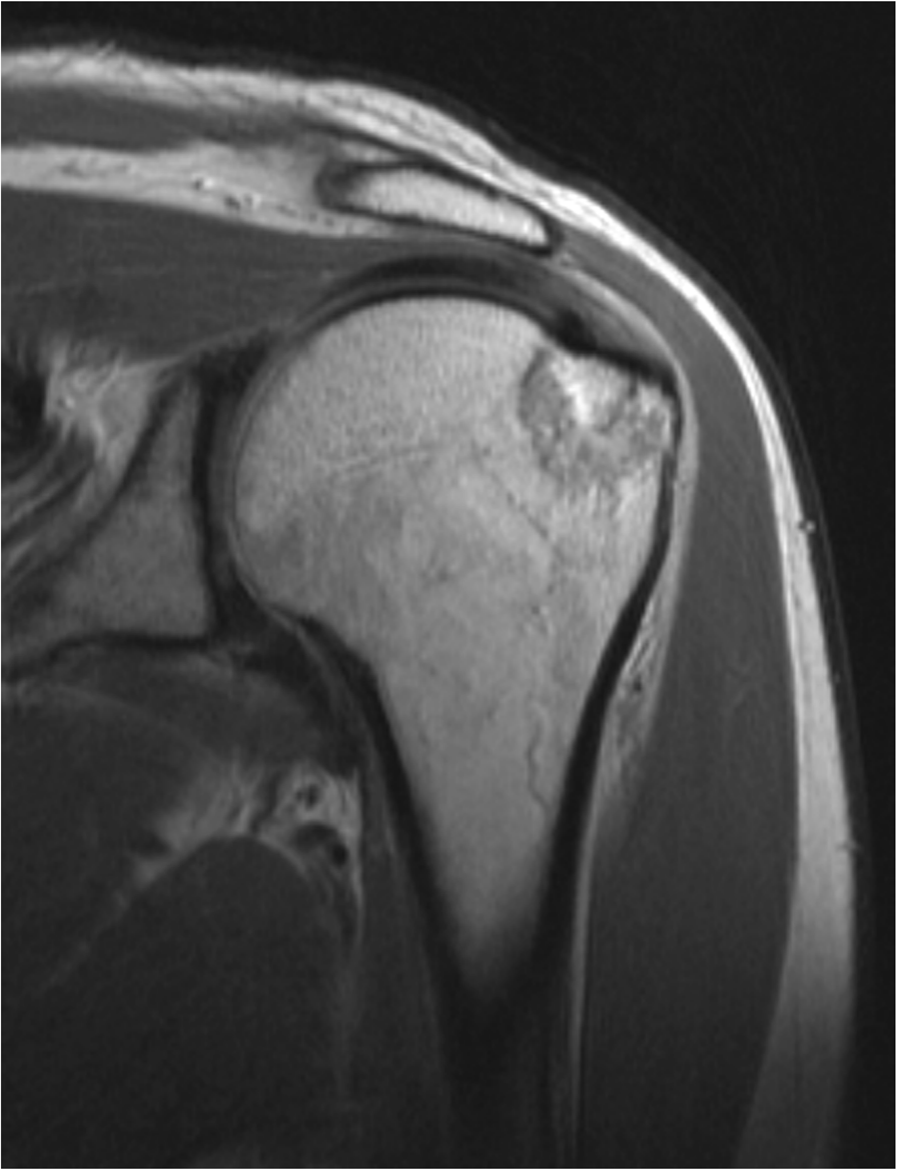
MRI showing a visible fracture line of undisplaced greater tuberosity due to bony reabsorption at the fracture line

Data on demographics, interval from initial injury to diagnosis, associated injury at MRI, type of primary injury, treatment and complications are reported in Table 1. Figures 1 and 2 depicts the typical radiographic and MRI findings in these patients.

Following initial treatment all fractures united with no secondary displacement. Three patients required glenohumeral joint injections for post traumatic stiffness between 6 and 12 weeks post injury and three patients required arthroscopy and subacromial decompression for post-traumatic impingement between 9 and 20 months post injury.

## Discussion

The main finding of this study is that in the majority of patients with a traumatic undisplaced fracture of the greater tuberosity, non-operative management was effective, allowing a safe and prompt return to activities.

Isolated fractures of the greater tuberosity account for approximately 20% of all proximal humeral fractures and are associated with a glenohumeral dislocation in approximately 10—30% of cases [2]. Kim et al. [8] reported that isolated greater tuberosity fractures occurs frequently in male patients with a mean age of 42.8 years. Moreover patients with isolated greater tuberosity fractures had fewer medical comorbidities than those with surgical neck fractures [8]. Tuberosity avulsion or fracture may occur after a fall onto an outstretched upper extremity due to an eccentric load applied by the attached rotator cuff on the tuberosity, often in the setting of a traumatic glenohumeral dislocation. The majority of greater tuberosity fractures are undisplaced, however the impingement of the tuberosity against the acromion or the impact against the anteroinferior glenoid during glenohumeral dislocation/subluxation could cause the inferior displacement of the tuberosity [8].

Non-displaced or minimally displaced (< 5 mm) fractures of the greater tuberosity are usually treated non operatively. Indications for surgery is a displacement > than 5 mm and take into account factor such as fracture characteristics and patient characteristics (age, comorbidities, extremity dominance, pre-injury shoulder and individual level of function, local bone quality). Surgery may be performed with an open techniques with a standard deltopectoral approach or through a deltoid splitting approach or with an arthroscopically assisted technique.

According to our experience is rare that, if the arm is placed at the patient’s side in a sling without pillow in slight internal rotation, an undisplaced fracture become displaced.

Also, we used validated questionnaire-based outcome measures.

Patients with painful abduction and external rotation after shoulder trauma with no abnormality on plain radiographs should be always considered as potentially to have sustained an undisplaced greater tuberosity fracture. A consistent clinical finding that may differentiate from a rotator cuff injury is tenderness laterally over the greater tuberosity. Poor quality of radiographs, lack of the external rotation view or lack of clinical experience could be causes of missed diagnosis. MR examination subsequently performed due to persisting symptoms, revealed the fracture in all patients. Therefore, MRI should be always performed in patients with persistent pain, bony tenderness and decreased range of motion despite negative plain radiographs. This can avoid missed diagnosis and a potential source of patient dissatisfaction satisfaction and litigation.

Prevalence of rotator cuff tear associated with occult fractures of the greater tuberosity was lower than reported in literature (2 of 17 patients in our series), even though our series is too small to draw definitive conclusions. The involvement of the rotator cuff in previous reports was found in 11 of 24 patients, and 7 of 25 patients. Further studies are needed to better understand the relationship of symptoms caused by trauma and the presence of partial tendon tears, with or without fracture [4, 9–12].

The majority of patients were asymptomatic at the final follow up. Therefore healing of undisplaced greater tuberosity fracture is reliably achieved with non-operative treatment and surgical intervention should be only considered in case of persisting pain and shoulder function.

Strengths of the present study are that 2 fully trained shoulder surgeons performed all the diagnosis and treatment, and that the follow up evaluations were performed by an independent assessor.

## Conclusions

In conclusion, undisplaced greater tuberosity fractures may be managed non-operatively with good results in the majority of cases. Nevertheless, shoulder MRI is warranted to confirm the diagnosis in patients with persistent post-traumatic shoulder pain and limitation of shoulder function with negative radiographs. Prompt identification of these fractures can facilitate patient management and information, particularly in counselling patients regarding the risk of stiffness and post-traumatic impingement. This will avoid a source of patient dissatisfaction and litigation.

## Abbreviations

AP: Anteroposterior
MRI: Magnetic Resonance Imaging
OSS: Oxford Shoulder Score
SD: Standard deviation
